# Eosinophilic Ascites: A Rare Presentation of a Hematoma

**DOI:** 10.7759/cureus.82712

**Published:** 2025-04-21

**Authors:** Mufaddal M Jafferjee, Morad H Amar, Vivek Ramarathnam, Shovendra Gautam

**Affiliations:** 1 Graduate Medical Education and Internal Medicine, Baylor Scott & White All Saints Medical Center, Fort Worth, USA; 2 Graduate Medical Education, Baylor Scott & White All Saints Medical Center, Fort Worth, USA; 3 Infectious Disease, Baylor Scott & White All Saints Medical Center, Fort Worth, USA; 4 Internal Medicine, Baylor Scott & White All Saints Medical Center, Fort Worth, USA

**Keywords:** ascites, eosinophilia, eosinophilic gastroenteritis, hes, liver hematoma

## Abstract

Idiopathic eosinophilic ascites (IEA) is a condition characterized by the eosinophilic infiltration of the peritoneal cavity without an identifiable underlying cause. We present the case of a 39-year-old female patient with no significant medical history who was referred for leukocytosis and abdominal discomfort. Imaging revealed a large, peripherally calcified intra-abdominal fluid collection, and subsequent paracentesis demonstrated eosinophilic ascites (EA). A thorough evaluation, including peripheral eosinophilia workup, malignancy screening (including imaging and cytology), infectious serologies (parasitic and fungal panels), autoimmune markers, and upper and lower endoscopy with biopsies, failed to identify a definitive etiology. Diagnostic laparoscopy confirmed a hematoma, and post-laparoscopy, the patient's symptoms and peripheral eosinophilia resolved. This case highlights the diagnostic challenges of EA, the importance of a systematic exclusionary approach, and the potential for an idiopathic, reactive eosinophilic process. Given the risk of recurrence or progression to an underlying disorder, long-term follow-up is warranted.

## Introduction

Ascites often appears as a manifestation of various diseases such as cirrhosis, heart failure, neoplasia, tuberculosis, or pancreatic disease [1]. The most common causes of ascites are parenchymal liver disease (78%) and malignancy (12%), followed by cardiovascular disease (5%) [2]. Eosinophilic ascites (EA) is an uncommon clinical entity defined by an eosinophilic predominance in ascitic fluid, often accompanied by peripheral eosinophilia. It can arise from a diverse range of etiologies, including parasitic infections, malignancies, hypereosinophilic syndrome (HES), and eosinophilic gastroenteritis (EGE). However, in some cases, no definitive cause is identified, leading to a diagnosis of idiopathic eosinophilic ascites (IEA).

Here, we present the case of a 39-year-old female patient with IEA and a concurrent large, calcified intra-abdominal fluid collection. Despite an exhaustive workup, no underlying cause was identified, and the patient's eosinophilia resolved post-laparoscopic exploration, suggesting a reactive process rather than a primary eosinophilic disorder. This case underscores the diagnostic complexity of EA and the importance of vigilant long-term monitoring for potential disease progression.

## Case presentation

**Figure 1:**
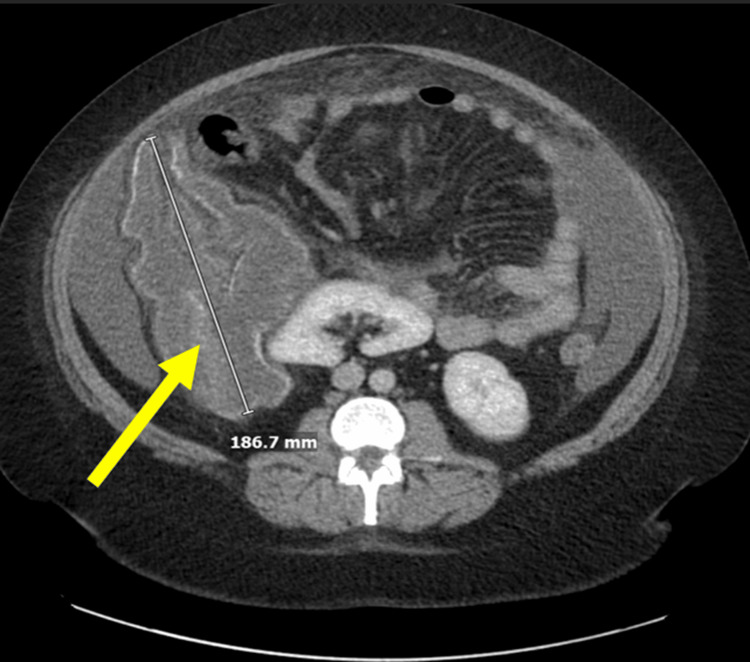
CT scan of the abdomen and pelvis showing a peripherally calcified, lobulated fluid collection (18.7 cm) inferior to the liver CT: computed tomography

A 39-year-old woman with no significant past medical history presented to the emergency department (ED) following a physician referral for leukocytosis. She reported mild generalized abdominal discomfort, cramping, and subjective fevers, with tachycardia but no fever on presentation. Laboratory evaluation revealed leukocytosis (28.9×10³/μL) with eosinophilic predominance (15.9×10³/μL, 55%) and serum immunoglobulin E levels of 541 kU/L (Table 1). A computed tomography (CT) scan of the abdomen and pelvis showed a peripherally calcified, lobulated fluid collection (18.7 cm) inferior to the liver (Figure 1) unchanged from a similar imaging study a month prior. Given concerns for intra-abdominal infection, she was admitted for further evaluation.

**Table 1:** Initial laboratory analysis on presentation WBC: white blood cell; IgE: immunoglobulin E

Parameter	Result	Reference range
WBC count	28.9×10³/μL	4.0-11.0×10³/μL
Eosinophil count	15.9×10³/μL	0.0-0.5×10³/μL
Eosinophil percentage	55%	<5%
IgE	541 kU/L	0-114 kU/L

A month earlier, she visited another ED after a fall, where imaging showed a large, irregular intra-abdominal fluid collection with peripheral calcifications. As she was asymptomatic and her workup was largely unremarkable, she was discharged with outpatient follow-up.

She subsequently underwent diagnostic and therapeutic paracentesis, yielding 5.5 liters of ascitic fluid. Analysis of the ascitic fluid showed 20,000/μL of red blood cells (RBCs) and 2006 nucleated cells/uL with eosinophilic predominance (76%), with a serum-ascitic albumin gradient (SAAG) <1.1, total protein >2.5 g/dL, lactate dehydrogenase (LDH) 450 U/L, glucose 63 mg/dL, and amylase 6 U/L. Cytologic analysis of the ascitic fluid was negative for malignancy, and histopathologic examination of the peritoneal biopsies revealed no evidence of malignancy. Microbiologic studies, including bacterial and fungal cultures as well as acid-fast and fungal stains, were all negative (Table 2).

**Table 2:** Laboratory analysis from initial paracentesis Additional tests (cytology, bacterial/fungal cultures, and acid-fast and fungal staining) were reported as negative and are not included in the table. RBCs: red blood cells; SAAG: serum-ascitic albumin gradient; LDH: lactate dehydrogenase

Parameter	Result	Reference range
RBCs	20000/μL	<1000/μL
Nucleated cells	2006/μL	<500/μL
Eosinophils (predominance)	76%	<10%
SAAG	<1.1	-
Total protein	>2.5	-
LDH	450 U/L	<225 U/L
Glucose	63 mg/dL	>50 mg/dL
Amylase	6 U/L	0-137 U/L

Concurrently, CT-guided aspiration and drainage of the intra-abdominal collection were performed, with fluid studies that showed 10,000 RBCs and 1145 nucleated cells/uL with eosinophilic predominance (51%). Acid-fast staining, cultures, and cytology of the aspirated fluid were also unremarkable. An extensive infectious workup, including serologies for *Echinococcus*, *Strongyloides*, and *Toxocara* (ELISA), as well as urine *Chlamydia trachomatis* PCR, *Neisseria gonorrhoeae* NAA, and the quantiferon TB test, was negative (Table 3).

**Table 3:** Summary of fluid analysis and infectious workup from CT-guided aspiration RBCs: red blood cells; CT: computed tomography

Parameter	Result	Reference range
RBCs	10000/μL	<1000/μL
Nucleated cells	1145/μL	<500/μL
Eosinophil percentage	51%	<10%
Acid-fast staining	Negative	-
Cultures (bacterial/fungal)	No growth	-
Cytology	Negative for malignant cells	-
*Echinococcus s*erology (ELISA)	Negative	-
*Strongyloides *serology (ELISA)	Negative	-
*Toxocara* serology (ELISA)	Negative	-
Urine *Chlamydia trachomatis* PCR	Negative	-
*Neisseria gonorrhoeae* NAA	Negative	-
Quantiferon TB test	Negative	-

Serum tumor markers, including carcinoembryonic antigen (CEA), carbohydrate antigen 19-9 (CA 19-9), cancer antigen-125 (CA-125), and alpha-fetoprotein (AFP), were within normal limits (Table 4).

**Table 4:** Serum tumor marker results CEA: carcinoembryonic antigen; CA 19-9: carbohydrate antigen 19-9; CA-125: cancer antigen-125; AFP: alpha-fetoprotein

Tumor marker	Result	Reference range
CEA	<2 ng/mL	<3 ng/mL
CA 19-9	5 U/mL	<37 U/mL
CA-125	15 U/mL	<35 U/mL
AFP	4.6 ng/mL	<10 ng/mL

She underwent diagnostic esophagogastroduodenoscopy (EGD) and colonoscopy, which were negative for any masses. Biopsies of the esophagus, stomach, duodenum, terminal ileum, and colon did not show any acute inflammatory infiltrates and were negative for atypia, dysplasia, or malignancy or eosinophilic infiltration.

**Figure 2:**
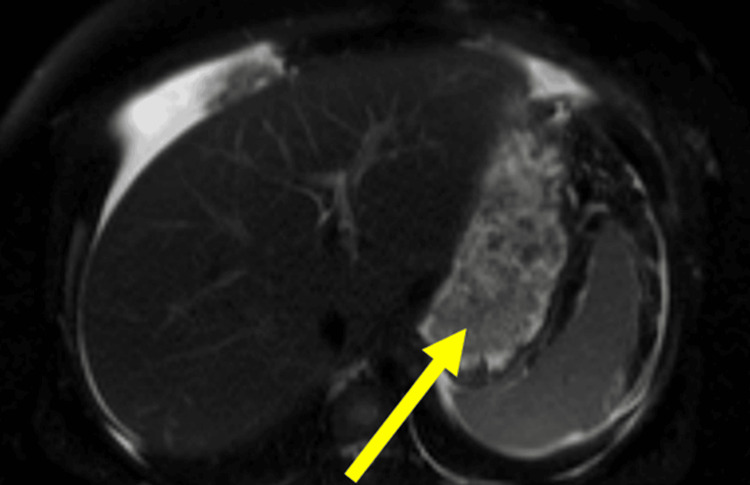
Follow-up MRI of the abdomen and pelvis showing persistent calcified intraperitoneal fluid collection (15.8×9.9×2 cm) despite aspiration and drainage of fluid collection seven days prior MRI: magnetic resonance imaging

Given the intra-abdominal collection in the right upper quadrant and unexplained ascites, a magnetic resonance imaging (MRI) of the abdomen and pelvis was obtained to evaluate for underlying liver pathology, which identified a thick-walled, calcified intraperitoneal fluid collection (15.8×9.9×2 cm) (Figure 2) and a simple hepatic cyst.

Due to the MRI findings, the patient underwent diagnostic laparoscopy with peritoneal and pelvic biopsies and repeat paracentesis for cytology. Laparoscopic exploration of the abdominal cavity revealed a moderate amount of ascitic fluid, which was aspirated for analysis. Multiple plaque-like lesions were observed on the peritoneum, particularly in the pelvis, and these were biopsied for histopathologic evaluation. There was no pathology noted in the gastrointestinal tract and liver. The known right upper quadrant cystic structure with a previously placed drain was seen. Pathology of both peritoneal and pelvic biopsies showed degenerating blood coagula containing cholesterol clefts with dense infiltrates of foamy histiocytes at the periphery, consistent with a diagnosis of organizing hematoma without any evidence of malignancy. Acid-fast and fungal stain, bacterial and fungal culture, and cytology of the aspirated ascitic fluid were all negative.

Secondary to initial concerns of bacterial infection at the time of admission, she completed an empiric 10-day course of piperacillin-tazobactam. By the end of the hospitalization and post-laparoscopic exploration day 2, she experienced symptomatic improvement with the normalization of leukocytosis and peripheral eosinophilia. Despite extensive evaluation, the etiology of her EA remained unclear. She was discharged with recommendations for the continued outpatient monitoring of eosinophilia and malignancy markers.

## Discussion

EA is a rare clinical manifestation characterized by the accumulation of eosinophil-rich fluid in the peritoneal cavity. It is defined by the presence of >100 eosinophils/µL or eosinophils comprising >10% of the non-erythrocyte count of the ascitic fluid [1].

In a systematic review of EA, of the patients with EA, 74% had EGE, 10% had parasitic or fungal infections, 7% had HES, and 9% had less common diseases, including eosinophilic pancreatitis, chronic eosinophilic leukemia, myelofibrosis, T-cell lymphoma, Churg-Strauss syndrome, familial paroxysmal polyserositis, and Ménétrier's disease [3].

In this case, a comprehensive diagnostic evaluation, including gastrointestinal endoscopy with targeted biopsies, tumor marker assessment, and infectious serologies, yielded no definitive etiology. The absence of gastrointestinal mucosal eosinophilic infiltration diminished the likelihood of EGE [4,5], while the lack of systemic organ involvement and persistent eosinophilia for greater than six months argued against a diagnosis of HES [6,7].

A salient feature of this case was the presence of a large, calcified intra-abdominal fluid collection initially presumed to be an abscess or neoplastic lesion and seen on CT of the abdomen during admission. Despite CT-guided interventional radiology (IR) drainage of the fluid collection, a follow-up MRI of the abdomen, which was done to evaluate for underlying liver pathology, showed a persistent calcified intraperitoneal fluid collection (15.8×9.9×2 cm) and a simple hepatic cyst. The intraperitoneal fluid collection later was found to be a hematoma upon laparoscopic evaluation, later confirmed with biopsy. Histopathologic analysis identifying a hematoma suggests a possible inflammatory response secondary to hemorrhage, potentially inciting an eosinophilic reaction.

The exact mechanism by which hematomas induce eosinophilic infiltration remains unclear. However, tissue injury and fibrin deposition could stimulate the release of cytokines such as interleukin-5 (IL-5) and eotaxin, both of which are known to promote eosinophil recruitment and activation [8]. Further studies are needed to determine the role of these mediators in eosinophilic peritoneal reactions. Notably, previous cases of EA secondary to intra-abdominal hematomas have not been reported in the literature. The absence of prior reports, coupled with the immunological mechanisms proposed in this case, underscores its novelty.

Prior literature has documented eosinophilic peritonitis in the context of peritoneal irritation, including hemorrhagic processes, peritoneal dialysis, and pharmacologic exposures [9,10]. The resolution of peripheral eosinophilia, ascites, and abdominal pain post-laparoscopic exploration and without the use of corticosteroid treatment, which is the mainstay therapy for EA, supports the hypothesis of a reactive, rather than primary, eosinophilic disorder. 

Given the idiopathic nature of this case, ongoing surveillance remains imperative to detect latent etiologies. Reports exist of EA preceding the diagnosis of progressing to chronic eosinophilic syndromes [4,11]. Serial eosinophil counts should be obtained every 3-6 months, with repeat abdominal imaging if symptoms recur and close monitoring for systemic involvement if persistent eosinophilia develops. Early corticosteroid intervention should be considered if progressive eosinophilic disease is suspected.

## Conclusions

This case underscores the inherent diagnostic challenges posed by EA and emphasizes the necessity of a rigorous exclusionary approach. Clinicians must maintain vigilance for both common and obscure etiologies, recognizing that idiopathic cases, although infrequent, do occur. Further elucidation of the pathophysiological mechanisms underlying eosinophilic peritoneal responses is critical to refining diagnostic and therapeutic paradigms in this rare condition.
